# The association between symptoms of developmental coordination disorder and neuropsychological characteristics in children with and without ADHD

**DOI:** 10.3389/fpsyt.2024.1441102

**Published:** 2024-07-25

**Authors:** Taeyeop Lee, Jongseok Lim, Seonok Kim, Jichul Kim, Kee Jeong Park, Yoo-Sook Joung, Hyo-Won Kim

**Affiliations:** ^1^ Department of Psychiatry, University of Ulsan College of Medicine, Asan Medical Center, Seoul, Republic of Korea; ^2^ Department of Clinical Epidemiology and Biostatistics, University of Ulsan College of Medicine, Asan Medical Center, Seoul, Republic of Korea; ^3^ Department of Psychiatry, Sungkyunkwan University School of Medicine, Samsung Medical Center, Seoul, Republic of Korea

**Keywords:** attention deficit hyperactivity disorder (ADHD), developmental coordination disorder (DCD), developmental coordination disorder questionnaire (DCDQ), cognitive profile, behavioral characteristics

## Abstract

**Objective:**

Attention-deficit/hyperactivity disorder (ADHD) frequently co-occurs with developmental coordination disorder (DCD). This study aimed to evaluate the association between DCD symptoms and neuropsychological characteristics in children with and without ADHD.

**Methods:**

We recruited 298 children aged 5–12 years. Motor performance was assessed using the Developmental Coordination Disorder Questionnaire (DCDQ), while ADHD symptoms were assessed using the ADHD Rating Scale (ARS) and the Advanced Test of Attention (ATA). Cognitive characteristics were measured using the Wechsler Intelligence Scale, and behavioral characteristics were assessed using the Korean Personality Rating Scale for Children.

**Results:**

The children had a mean age of 7.6 ± 1.7 years, with 214 (71.8%) being boys. Among children diagnosed with ADHD (n = 176), 39.2% exceeded the DCDQ cutoff score, compared to 4.1% in the neurotypical group (n = 122). In the correlation analysis, the DCDQ total score was significantly correlated with ARS, omission and commission errors in visual and auditory ATA, and full-scale intellectual quotient. In addition, symptoms of depression, social dysfunction, and psychosis were correlated with the DCDQ total score. In the between-group analysis, children with both ADHD and DCD exhibited more omission errors on the auditory ATA and behavioral problems related to depression, social dysfunction, and psychosis compared to children with ADHD only.

**Conclusion:**

Our study indicates that children with ADHD exhibit more difficulties in motor performance. Children with both ADHD and DCD may present with a greater burden of psychiatric conditions than children with ADHD only, suggesting the need for careful monitoring in clinical practice.

## Introduction

Attention-deficit/hyperactivity disorder (ADHD) is a neurodevelopmental disorder characterized by inattention, hyperactivity, and impulsivity. ADHD is common, with prevalence estimates of 5–7% in children and adolescents (1, 2). ADHD often co-occurs with other psychiatric disorders, with approximately 70–80% of individuals having a comorbid condition (3). These comorbidities are important in that they influence symptom presentation, complicate treatment, and predict long-term outcomes.

Among the common comorbidities of ADHD is developmental coordination disorder (DCD), which is classified as a neurodevelopmental disorder (4). DCD manifests as difficulties in fine and gross motor skills, significantly impacting daily activities and functioning both in school and home settings (5). Tasks such as using scissors, buttoning clothing, catching balls, and climbing stairs are challenging for children with DCD. With prevalence estimates ranging from 2–5%, DCD is a prevalent condition that often co-occurs with ADHD (6, 7).

The co-occurrence rate of ADHD and DCD is high, with approximately half of children diagnosed with ADHD meeting the criteria for DCD (8, 9). While the concept of attention deficits, motor control, and perception was introduced in the 1970s (10), motor difficulties were not regarded as a distinct condition from ADHD symptoms (11). Consequently, the co-occurrence of ADHD and DCD has been relatively understudied, and debates continue regarding their neurobiological similarities and differences (12). Furthermore, existing literature comparing the neuropsychological characteristics of children with ADHD with and without comorbid DCD is limited by small sample sizes, thus warranting further investigation (13).

In this study, our primary objective was to explore the association between DCD symptoms and neuropsychological characteristics in children with and without ADHD. Specifically, we aimed to examine the correlation between motor performance and neuropsychological profiles, and to compare the neuropsychological characteristics between children with ADHD with DCD, children with ADHD without DCD, and neurotypical children. By addressing these objectives, we aimed to contribute to understanding the interplay between ADHD and DCD.

## Methods

### Study participants

A total of 298 children aged 5–12 years participated in this study, including 176 children diagnosed with ADHD and 122 neurotypical children. The children were enrolled between May 2014 and May 2020. Children with ADHD were recruited at the Children’s Hospital of Asan Medical Center, located in Seoul, Republic of Korea. ADHD was diagnosed by board-certified child and adolescent psychiatrists according to the fourth and fifth editions of the Diagnostic and Statistical Manual of Mental Disorders (DSM-IV and DSM-5) and the Kiddie–Schedule for Affective Disorders and Schizophrenia–Present and Lifetime Version (K-SADS-PL). Neurotypical children were recruited through advertisements in the hospital and on internet sites. Neurotypical children were screened for any psychiatric disorder according to the DSM-IV/DSM-5 and K-SADS-PL. Children were excluded from the study if they had a Full-Scale Intellectual Quotient (FSIQ) lower than 70, as confirmed by the Wechsler Intelligence Scale for Children, when they were diagnosed with autism spectrum disorder, schizophrenia, organic mental disorder, or neurologic disorders such as epilepsy. Informed consent was obtained from the parents or guardians of the study participants, and assent was obtained from the study participants, where appropriate. The study was performed in line with the principles of the Declaration of Helsinki and was approved by the Institutional Review Board of Asan Medical Center (2014-0157).

### Clinical assessment

#### The developmental coordination disorder questionnaire

The DCDQ is a questionnaire that evaluates the motor performance of a child (14). DCDQ is administered by parents or caregivers and involves a comparison of the child’s motor abilities with those of other children in the same age group. The questionnaire assesses three sub-components of motor performance, which are control during movement, fine motor/handwriting, and general coordination. The DCDQ is composed of 15 items, which are rated on a 5-point Likert scale. Children are classified as indication of DCD or probably not DCD, according to their age and total score (indication of DCD: total score ≤46 for age 5:0–7:11 years; total score ≤55 for age 8:0–9:11 years; and total score ≤57 for age 10:0–15:11 years).

#### ADHD rating scale

The severity of ADHD symptoms was assessed using the ARS, completed by parents or caregivers. The ARS is designed to evaluate ADHD symptoms in school-age children and consists of 18 items divided into two subscales: inattention and hyperactivity/impulsivity. The validity and reliability of the scale have been well established (15).

#### Korean personality rating scale for children

Behavioral characteristics of children and adolescents were evaluated using the KPRC, which was completed by parents or caregivers (16). The KPRC is an adapted version of the Personality Inventory for Children and comprises 177 items categorized into 10 subscales, namely verbal development delay, physical development delay, anxiety, depression, somatization, delinquency, hyperactivity, family dysfunction, social dysfunction, and psychosis. This scale was previously standardized in a sample of 2,639 children and adolescents in Korea, and its reliability and validity have been established (16).

#### Wechsler intelligence scale for children–fourth edition and Wechsler preschool and primary scale of intelligence–fourth edition

Intelligence was evaluated using the WISC-IV and WPPSI-IV (17, 18), which were administered by clinical psychologists. The WISC-IV is used in children aged 6:0–16:11 years and includes four primary indices: verbal comprehension, perceptual reasoning, working memory, and processing speed. The WPPSI-IV is used in children aged 2:6–7:7 years and includes five primary indices: verbal comprehension, visual-spatial, fluid reasoning, working memory, and processing speed. The FSIQ is derived from the four or five primary indices. This widely used test exhibits good to excellent internal consistency. For the primary indices analysis, only results from the WISC-IV were used (n = 255).

#### Advanced test of attention

The neuropsychological profile of attention was examined using the ATA, administered by clinical psychologists (19). ATA is a computerized continuous performance test, and four major variables, including omission errors, commission errors, response time, and response time variability, are collected and transformed into Z-scores. The ATA was standardized in the Korean population, and the psychometric properties of the test have been established (19).

### Statistical analysis

The ADHD and neurotypical groups were compared for demographic characteristics and motor performance. Categorical variables were compared using a chi-square test, and continuous variables were compared using a student’s t-test. Correlations between DCDQ scores and neuropsychological measures were assessed using the Pearson correlation method. ANOVA was used to compare the three groups, namely the “ADHD with DCD,” “ADHD without DCD,” and “neurotypical” groups. When a significant difference existed among the three groups, a *post-hoc* Bonferroni test for multiple comparisons was performed to evaluate the differences between the groups. For the estimation of effect sizes, eta squared (η^2^) values were calculated. Data analyses were performed using R Statistical Software, version 4.0.2 (R Foundation for Statistical Computing, Vienna, Austria).

## Results

The demographic characteristics of the children who participated in this study are summarized in **Table 1**.** A** total of 298 children participated in the study (age 7.6 ± 1.7 years old, 71.8% boys). The ADHD and neurotypical groups did not show significant differences in age and familial income. However, compared to the neurotypical group, the ADHD group consisted predominantly of boys, and were more often diagnosed with tic disorder. The education level of fathers of children in the ADHD group showed a significant difference compared to the neurotypical group.

**Table 1:** Summary sample characteristics.

Variables	ADHD (n = 176)	Neurotypical (n = 122)	*p*
Age, mean (SD), month	7.5 (2.1)	7.6 (1.5)	0.628
Sex, n (%)			
Boys	152 (87.9%)	62 (50.8%)	<0.001
Presentation of ADHD diagnosis, n (%)			
Inattentive	60 (34.1%)	–	
Hyperactive/impulsive	4 (2.3%)	–	
Combined	95 (54.0%)	–	
Unspecified	17 (9.7%)	–	
Comorbid diagnosis, n (%)			
ODD	17 (9.7%)	0 (0%)	0.001
SAD	1 (0.6%)	2 (1.6%)	0.748
Social phobia	3 (1.7%)	1 (0.8%)	0.888
Specific phobia	5 (2.8%)	3 (2.5%)	1.000
GAD	1 (0.6%)	0 (0%)	1.000
Enuresis	4 (2.3%)	0 (0%)	0.244
Tic disorder	24 (13.7%)	5 (4.1%)	0.011
Family income, n (%)			
Low	19 (10.8%)	5 (4.1%)	0.109
Middle	70 (40%)	50 (41.3%)	
High	86 (49.1%)	66 (54.5%)	
Education, father, n (%)			
High school or less	33 (18.9%)	4 (3.3%)	<0.001
Bachelor’s degree	117 (66.9%)	106 (87.6%)	
Graduate school	25 (14.3%)	11 (9.1%)	
Education, mother, n (%)			
High school or less	30 (17.1%)	9 (7.4%)	0.050
Bachelor’s degree	121 (69.1%)	95 (78.5%)	
Graduate school	24 (13.7%)	17 (14%)	

ADHD, Attention Deficit Hyperactivity Disorder; GAD, Generalized Anxiety Disorder; ODD, Oppositional Defiant Disorder; SAD, Separation Anxiety Disorder; SD, Standard Deviation.

We compared the motor performance of the ADHD and neurotypical groups as evaluated by the DCDQ (**Table 2**). In the ADHD group, 39.2% (n = 176) of the children were classified as indication of DCD, whereas in the neurotypical group, 4.1% (n = 5) were classified as indication of DCD. The DCDQ total score and the three sub-component scores were significantly lower in the ADHD group than in the neurotypical group, indicating more difficulties in motor performance.

**Table 2:** Comparison of motor skills among ADHD and neurotypical groups.

Variables	ADHD (n = 176)	Neurotypical (n = 122)	*p*
DCDQ cutoff, n (%)	69 (39.2%)	5 (4.1%)	<0.001
DCDQ, mean (SD)			
Total score	52.8 (8.2)	59.4 (11)	<0.001
Control during movement	23.0 (3.6)	25.0 (5)	<0.001
Fine motor/handwriting	14.7 (2.4)	17.8 (3.7)	<0.001
General coordination	15.1 (4.1)	16.6 (4.2)	0.008

DCDQ, Developmental Coordination Disorder Questionnaire.

**Figure 1:**
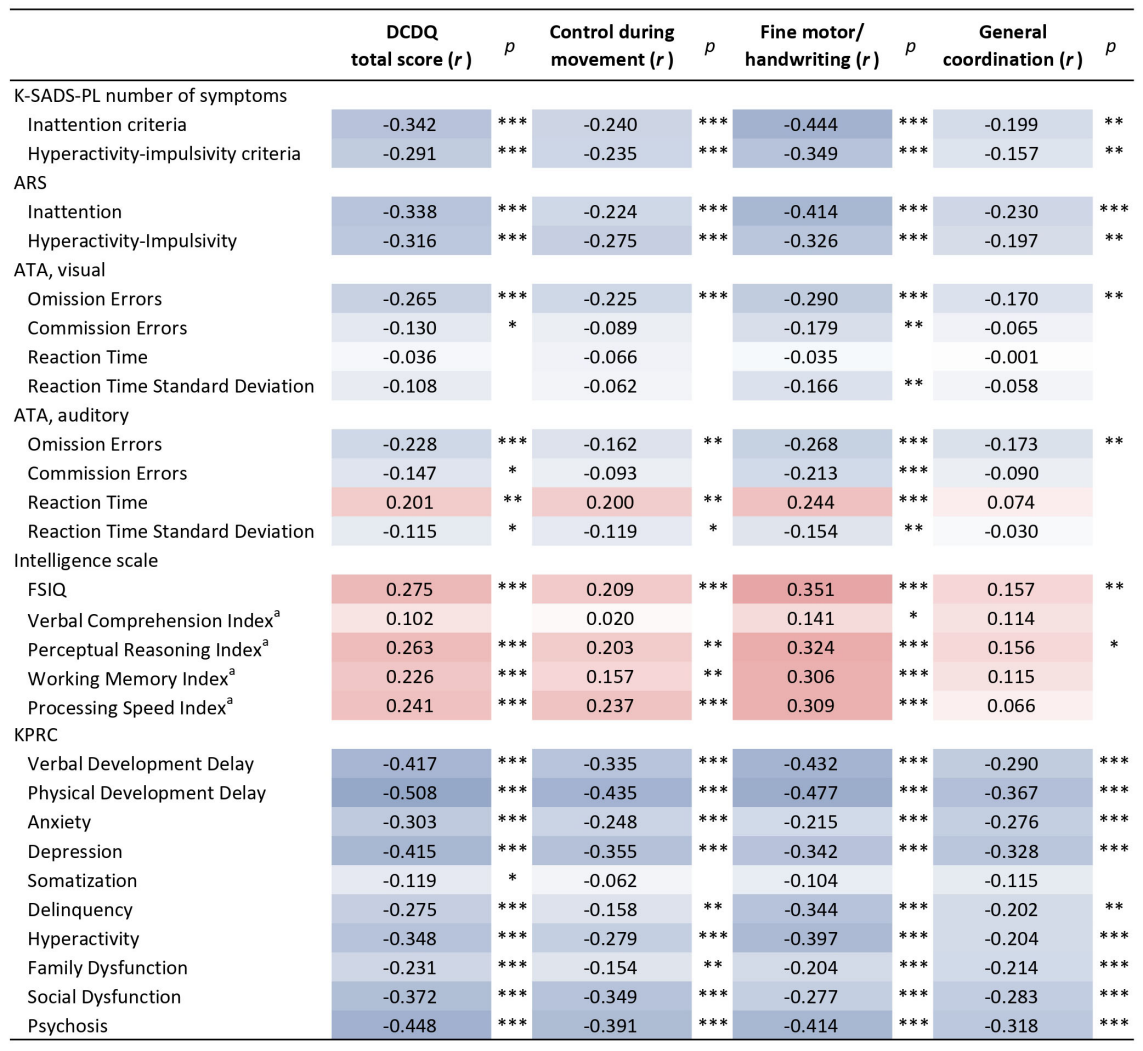
Correlation between DCDQ scores and neuropsychological measures. Positive correlations are displayed in red and negative correlations in blue. The color shade corresponds to the correlation coefficients, with darker shades indicating stronger correlations. *p<0.05; **p<0.01, ***p<0.001; ARS, ADHD Rating Scale; ATA, Advanced Test of Attention; DCDQ, Developmental Coordination Disorder Questionnaire; FSIQ, Full Scale Intelligence Quotient; KPRC, Korean Personality Rating Scale for Children; K-SADS-PL, Kiddie-Schedule for Affective Disorders and Schizophrenia-Present and Lifetime Version. ^a^Primary indices from the WISC-IV were used (n = 255).

The correlation between DCDQ scores and neuropsychological measures was examined (**Figure 1**). The DCDQ total score was significantly correlated with ADHD symptoms measured by K-SADS-PL and ARS. Specifically, the DCDQ total score showed a negative correlation with both inattentive symptoms (K-SADS-PL, r = -0.342; ARS, r = -0.338) and hyperactivity-impulsivity symptoms (K-SADS-PL, r = -0.291; ARS, r = -0.316). The DCDQ total score showed a higher correlation with inattentive symptoms than with hyperactivity-impulsivity symptoms. In addition, the DCDQ total score showed a significant correlation with omission and commission errors of visual ATA (r = -0.265 and r = -0.130, respectively), and omission errors, commission errors, reaction time, and reaction time standard deviation of auditory ATA (r = -0.228, r = -0.147, r = 0.201, and r = -0.115, respectively). The omission errors of both visual and auditory ATA showed the largest extent of correlation with the DCDQ total score, compared to that with other ATA variables. The DCDQ total score showed a significant correlation with FSIQ (r = 0.275) and perceptual reasoning, working memory, and processing speed indices (r = 0.263, r = 0.226, and r = 0.241, respectively). Behavioral characteristics were examined with KPRC, where all subscales showed a significant correlation with the DCDQ total score. Of the subscales, physical development delay, psychosis, verbal development delay, and depression showed relatively high correlation coefficients (r = -0.508, r = -0.448, r = -0.417, and r = -0.415, respectively).

The correlation between DCDQ sub-components and neuropsychological measures was examined (**Figure 1**). Among the three sub-components, fine motor/handwriting exhibited the largest extent of correlation with a majority of the measures, except for the subscales of KPRC, including anxiety, depression, family dysfunction, and social dysfunction.

Next, we assessed whether neuropsychological characteristics differed according to the presence of DCD in the ADHD group (**Table 3**). To this end, we compared the “ADHD with DCD,” “ADHD without DCD,” and “neurotypical” groups. Compared to the “ADHD without DCD” group, the “ADHD with DCD” group showed significantly higher omission error scores on auditory ATA and higher verbal development delay, physical development delay, depression, social dysfunction, and psychosis subscale scores on KPRC.

**Table 3:** Comparison of neuropsychological measures among the ADHD with DCD, ADHD without DCD, and neurotypical groups.

	ADHD with DCD (n = 69)	ADHD without DCD (n = 107)	Neurotypical (n = 122)	F	η^2^	*p*
	Mean (SD)	Mean (SD)	Mean (SD)			
K-SADS-PL number of symptoms						
Inattention criteria	7.5 (1.3)	7.2 (1.4)	0.9 (1.4)	791.1	0.845	<0.001^b,c^
Hyperactivity-impulsivity criteria	5.2 (2.7)	5.8 (2.3)	0.7 (1.2)	209.4	0.591	<0.001^b,c^
ARS						
Inattention	14.6 (5.2)	13.3 (5.5)	3.6 (3.0)	180.5	0.550	<0.001^b,c^
Hyperactivity-impulsivity	11.0 (5.7)	11.0 (5.7)	2.4 (2.4)	122.2	0.453	<0.001^b,c^
ATA, visual						
Omission errors	5.0 (4.9)	3.9 (5.2)	1.0 (2.8)	22.6	0.134	<0.001^b,c^
Commission errors	3.0 (3.3)	4.1 (4.1)	1.3 (2.4)	20.6	0.124	<0.001^b,c^
Reaction time	1.2 (1.7)	0.7 (1.6)	0.9 (1.3)	2.7	0.018	0.069
Reaction time standard deviation	2.4 (2.5)	2.8 (3.2)	0.8 (2.5)	15.3	0.095	<0.001^b,c^
ATA, auditory						
Omission errors	2.0 (2.8)	1.1 (1.9)	0.2 (1.5)	18.1	0.111	<0.001^a,b,c^
Commission errors	2.0 (2.7)	2.2 (2.2)	0.4 (1.8)	22.9	0.136	<0.001^b,c^
Reaction time	-0.8 (1.5)	-1.0 (1.5)	0.0 (0.9)	19.3	0.117	<0.001^b,c^
Reaction time standard deviation	0.1 (1.2)	0.5 (1.1)	-0.4 (0.9)	19.0	0.116	<0.001^b,c^
Intelligence scale						
FSIQ	93.3 (15.0)	95.8 (14.2)	109.5 (13.5)	39.1	0.211	<0.001^b,c^
Verbal comprehension index	101.7 (13.6)	98.9 (11.0)	107.7 (12.3)	12.3	0.089	<0.001^b,c^
Perceptual reasoning index	99.2 (16.7)	99.5 (16.0)	109.1 (15.7)	11.5	0.084	<0.001^b,c^
Working memory index	92.0 (14.3)	93.8 (15.3)	109.1 (13.8)	38.0	0.232	<0.001^b,c^
Processing speed index	84.9 (14.4)	88.8 (13.5)	99.3 (13.7)	25.4	0.168	<0.001^b,c^
KPRC						
Verbal development delay	60.2 (11.7)	52.6 (11.7)	45.3 (9.1)	42.6	0.228	<0.001^a,b,c^
Physical development delay	61.5 (10.0)	51.2 (10.3)	43.7 (10.3)	65.7	0.312	<0.001^a,b,c^
Anxiety	54.6 (10.0)	51 (11.0)	46.2 (11.2)	13.5	0.086	<0.001^b,c^
Depression	58.8 (10.8)	52.4 (10.4)	46.6 (9.8)	31.3	0.178	<0.001^a,b,c^
Somatization	47.5 (9.8)	46.3 (10.2)	42.6 (8.6)	7.1	0.047	0.001^b,c^
Delinquency	59.5 (11.6)	60.2 (12.7)	44.7 (9.5)	64.5	0.309	<0.001^b,c^
Hyperactivity	65.5 (11.5)	63.8 (11.6)	43.4 (9.5)	136.5	0.486	<0.001^b,c^
Family dysfunction	54.3 (13.2)	54.4 (14.0)	45.6 (10.1)	18.0	0.111	<0.001^b,c^
Social dysfunction	54.9 (12.0)	49.8 (10.1)	46.6 (9.7)	13.8	0.087	<0.001^a,b,c^
Psychosis	63.0 (12.2)	55.1 (12.2)	45.5 (9.6)	55.2	0.276	<0.001^a,b,c^

ATA, Advanced Test of Attention; DCD, Developmental Coordination Disorder; FSIQ, Full Scale Intelligence Quotient; KPRC, Korean Personality Rating Scale for Children; K-SADS-PL, Kiddie-Schedule for Affective Disorders and Schizophrenia-Present and Lifetime Version; SD, Standard Deviation.

a Significant difference between ADHD with DCD and ADHD without DCD.

b Significant difference between ADHD with DCD and neurotypical children.

c Significant difference between ADHD without DCD and neurotypical children.

## Discussion

In this study, we observed that DCD is nine times more prevalent in children with ADHD when compared to neurotypical children. The ADHD group exhibited lower DCDQ total and subscale scores than that in neurotypical children, indicating more difficulty in overall motor performance. In the correlation analysis, the DCDQ total score exhibited a significant correlation with ADHD symptoms and omission and commission errors in the continuous performance test and FSIQ. In addition, symptoms of depression, social dysfunction, and psychosis were correlated with the DCDQ total score. In between-group comparisons, the “ADHD with DCD” group showed higher levels of omission errors in the auditory continuous performance test than that in the “ADHD without DCD” group. Similarly, when assessing behavioral characteristics, the “ADHD with DCD” group exhibited higher scores on subscales related to verbal and physical development delay, depression, social dysfunction, and psychosis than the “ADHD without DCD” group.

The prevalence of DCD, based on the DCDQ cutoff score, was 39.2% in the ADHD group, compared to 4.1% in the neurotypical group. Previous studies conducted in Europe, Canada, and Australia, reported 30–50% prevalence of DCD among children with ADHD (20–22). These findings are comparable to those of our study conducted in South Korea, suggesting that the prevalence rate is consistent across different regions. Analysis of the DCDQ subscales, which assess control during movement, fine motor/handwriting, and general coordination, revealed significantly lower scores in the ADHD group than those in the neurotypical group, indicating deficits across various aspects of motor performance. Of these subscales, fine motor/handwriting exhibited the strongest correlation with ADHD symptoms. Given that complex motor activities necessitate higher-order cognitive skills, including executive function (23), fine motor/handwriting may require greater attentional resources and consequently show a stronger correlation with ADHD symptoms compared to other motor subscales.

In our analysis, we observed that among ADHD symptoms, inattention exhibited a stronger correlation with overall motor performance compared to hyperactivity/impulsivity. This observation highlights the significance of the inattentive symptoms in DCD. Children with DCD commonly present impairments in attention (24), working memory (25), and planning (26). However, the underlying mechanism driving the correlation between inattention and motor coordination remains unclear. One proposed explanation involves impaired inhibitory control, which may lead to reduced ability in action planning and motor learning (27). Another plausible model is the cognitive-energetic model, which emphasizes deficits in information processing (11). According to this model, encoding difficulties could contribute to attentional deficits in DCD. Our results indicate that symptoms of inattention, measured by clinicians, parents, and omission errors in the continuous performance test, are closely related to motor performance.

Motor performance exhibited a significant correlation with intelligence, as measured by FSIQ. The relationship between motor skills and cognitive function has long been recognized, tracing back to the sensorimotor stage of Piaget’s cognitive development theory (28). The execution of new motor skills facilitates cognitive processes in infants and children, enabling them to explore new aspects of their environment, thereby shaping their perceptions, particularly those related to spatial abilities and executive functions (29, 30). In our study, perceptual reasoning, working memory, and processing speed were correlated with motor performance, while verbal comprehension was not. This result is in concordance with previous studies (31, 32), where verbal comprehension, reflecting crystallized aspects of intelligence, did not exhibit a significant association with motor performance. Considering the association between motor performance and verbal development in children aged 3–6 years (33, 34), further studies are warranted to evaluate the role of age as a moderator in the relationship between motor performance and verbal development.

Children in the “ADHD with DCD” group exhibited more symptoms related to depression, social dysfunction, and psychosis than those in the “ADHD without DCD” and “neurotypical” groups. Studies addressing psychiatric problems in children with ADHD with and without DCD are limited and have primarily been conducted in Western countries. Missiuna et al. (35) compared psychological distress in adolescents with ADHD + DCD, ADHD alone, DCD alone, and in neurotypical children. In adolescents with ADHD + DCD, the prevalence of depression was 15 times higher than that in neurotypical children, and 3 to 5 times higher than that in adolescents with DCD or ADHD only. Another study with a smaller sample size reported increased peer victimization and emotional problems in the ADHD + DCD group compared to those in the DCD-only group (36). To the best of our knowledge, no study has compared psychosis-like behavior in children with ADHD with and without DCD. Considering the age group of the children enrolled in our study and the nature of the questions used in the KPRC, a higher score on the psychosis subscale suggests the presence of odd and eccentric behaviors, rather than true psychotic symptoms such as hallucination and delusion. Altogether, these findings underscore the heightened severity of behavioral problems in children with both ADHD and DCD, implying that psychiatric comorbidity needs to be carefully monitored.

This study has certain limitations. First, the children who participated in the study were recruited from a single hospital, limiting the generalizability of the findings to a broader population. Nonetheless, the consistency in diagnosis and measures applied across participants may enhance the reliability of the results. Second, while statistically significant, the correlations observed in the correlation analysis were at best moderate, if not weak (37). This may explain why most of the variables that show a significant correlation with motor performance did not demonstrate significant differences in the three-group comparison analysis between the ADHD groups with and without DCD and the neurotypical group. Third, the definition of DCD for group comparison relied on the DCDQ, which may lead to inaccurate group classification and potentially impact the study outcomes. Finally, considering the six-year enrollment period, the sample size of the study is modest.

In conclusion, we explored motor performance using the DCDQ in 298 children aged 5–12 years. Among children diagnosed with ADHD, 39.2% exceeded the cutoff scores of the DCDQ, contrasting with 4.1% in the neurotypical group. When compared to children with ADHD only, those with ADHD and DCD exhibited more omission errors on the auditory continuous performance test, along with behavioral problems related to depression, social dysfunction, and psychosis. Our study indicates that children with both ADHD and DCD may present with a greater burden of psychiatric conditions that need careful monitoring in clinical practice.

## Data availability statement

The raw data supporting the conclusions of this article will be made available by the authors, without undue reservation.

## Ethics statement

The study was approved by the Institutional Review Board of Asan Medical Center (2014-0157). The studies were conducted in accordance with the local legislation and institutional requirements. Written informed consent for participation in this study was provided by the participants’ legal guardians/next of kin.

## Author contributions

TL: Data curation, Formal analysis, Visualization, Writing – original draft, Writing – review & editing. JL: Data curation, Project administration, Writing – review & editing. SK: Formal analysis, Software, Writing – review & editing. JK: Data curation, Project administration, Writing – review & editing. KP: Data curation, Project administration, Writing – review & editing. Y-SJ: Conceptualization, Supervision, Writing – review & editing. H-WK: Conceptualization, Funding acquisition, Resources, Supervision, Writing – review & editing.
